# Supplementary material for: Land use/land cover changes in the central part of the Chitwan Annapurna Landscape, Nepal
Source: PeerJ. 2022 May 20;10:e13435. doi: 10.7717/peerj.13435 (PMC9126145; doi:10.7717/peerj.13435)
Supplement: Supplemental Information 1 [file peerj-10-13435-s001.pdf]

Supplementary Files

Supplementary Tables

Table S1 Different bands of Landsat 5 TM, Landsat 7 (ETM) and Landsat 8 (OLI) used for Band Combination.

Landsat 5 TM			Landsat 7 (ETM)			Landsat 8 OLI		
**Bands**	Wave length (μm)	Resolution	Bands	Wave length (μm)	Resolution	Bands	Wave length (μm)	Resolution
**Band 1- Blue**	0.45-0.52	30	Band 1- Blue	0.45-0.52	30	Band 1- Coastal aerosol	0.43-0.45	30
**Band 2- Green**	0.52-0.60	30	Band 2- Green	0.52-0.60	30	Band 2- Blue	0.45-0.51	30
**Band 3- Red**	0.63-0.69	30	Band 3- Red	0.63-0.69	30	Band 3- Green	0.53-0.59	30
**Band 4- Near Infrared Red (NIR)**	0.77-0.90	30	Band 4- Near Infrared Red (NIR)	0.77-0.90	30	Band 4- Red	0.64-0.67	30
**Band 5- SWIR1**	1.55-1.75	30	Band 5- SWIR1	1.55-1.75	30	Band 5- Near Infrared Red (NIR)	0.85-0.88	30
**Band 6- Thermal infrared (TIR)**	10.40-12.50	120	Band 6- Thermal infrared (TIR)	10.40-12.50	30/60	Band 6-SWIR1	1.57-1.65	30
**Band 7- SWIR2**	2.08-2.35	30	Band 7- SWIR2	2.09-2.35	30	Band 7- SWIR2	2.11-2.29	30
			Band 8- Panchromatic (Pan)	0.52-0.90	15	Band 8- Panchromatic (Pan)	0.50-0.68	15
						Band 9- Cirrus	1.36-1.38	30
						Band 10- Thermal infrared (TIRS1)	10.6-11.19	100
						Band 11- Thermal infrared (TIRS2)	11.5-12.51	100
