# Supplementary material for: Land use/land cover changes in the central part of the Chitwan Annapurna Landscape, Nepal
Source: PeerJ. 2022 May 20;10:e13435. doi: 10.7717/peerj.13435 (PMC9126145; doi:10.7717/peerj.13435)
Supplement: Supplemental Information 2 [file peerj-10-13435-s002.pdf]

Table S2 Land cover change from 2000 to 2020 in Old Padampur, New Padampur, Byas, Panchase Protected forest area, a part of ACA.

Land cover type	Area (km^2)			Change in area					
	2000	2010	2020	2000-2010		2010-2020		2000-2020	
				Area (km^2)	%	Area (km^2)	%	Area (km^2)	%
**Old Padampur area**									
**Water bodies**	3.48	3.31	2.99	-0.17	-4.88	-0.32	-9.66	-0.49	-14.08
**Barren area**	3.08	1.26	0.78	-1.82	-59.09	-0.48	-38.09	-2.3	-74.67
**Grassland**	7.94	17.31	15.44	9.37	118.01	-1.87	-10.8	7.5	94.45
**Riverine forest**	7.21	11.14	13.79	3.93	54.5	2.65	23.78	6.58	91.26
**Sal dominated forest**	1.08	1.13	1.16	0.05	4.62	0.03	2.65	0.08	7.4
**Mixed forest**	0.08	0.12	0.13	0.04	50	0.01	8.33	0.05	62.5
**Crop land**	12.19	0.86	0.81	-11.33	-92.94	-0.05	-5.81	-11.38	-93.35
**Developed area**	0.31	0.24	0.27	-0.07	-22.58	0.03	12.5	-0.04	-12.9
**New Padampur area**									
**Water bodies**	0.34	0.45	0.53	0.11	32.35	0.08	17.77	0.19	55.88
**Barren area**	0.68	0.2	0.3	-0.48	-70.58	0.1	50	-0.38	-55.88
**Grassland**	0.91	0.63	0.32	-0.28	-30.76	-0.31	-49.2	-0.59	-64.83
**Riverine forest**	11.86	4.53	4.6	-7.33	-61.8	0.07	1.54	-7.26	-61.21
**Sal dominated forest**	8.61	4.33	3.95	-4.28	-49.7	-0.38	-8.77	-4.66	-54.12
**Mixed forest**	1.1	0.41	1.15	-0.69	-62.72	0.74	180.48	0.05	4.54
**Crop land**	7.78	18.5	14.64	10.72	137.78	-3.86	-20.86	6.86	88.17
**Developed area**	0.39	2.42	5.98	2.03	520.51	3.56	147.107	5.59	1433.33
**Byas area**									
**Water bodies**	1.11	1.37	1.35	0.26	23.42	-0.02	-1.45	0.24	21.62
**Barren area**	0.73	0.82	0.75	0.09	12.32	-0.07	-8.53	0.02	2.73
**Grassland**	0.21	0.24	0.23	0.03	14.28	-0.01	-4.16	0.02	9.52
**Riverine forest**	0.04	0.05	0.05	0.01	25	0	0	0.01	25
**Sal dominated forest**	2.41	2.51	2.61	0.1	4.14	0.1	3.98	0.2	8.29
**Mixed forest**	3.17	3.59	5.14	0.42	13.24	1.55	43.17	1.97	62.14
**Crop land**	9.96	8.79	5.89	-1.17	-11.74	-2.9	-32.99	-4.07	-40.86
**Developed area**	1.86	2.12	3.47	0.26	13.97	1.35	63.67	1.61	86.55
**Panchase and associated area**									
**Water bodies**	2.57	2.49	2.5	-0.08	-3.11	0.01	0.401	-0.07	-2.72
**Barren area**	4.72	3.15	1.69	-1.57	-33.26	-1.46	-46.34	-3.03	-64.19
**Grassland**	5.02	3.23	2.85	-1.79	-35.65	-0.38	-11.76	-2.17	-43.22
**Riverine forest**	0.48	0.43	0.44	-0.05	-10.41	0.01	2.32	-0.04	-8.33

**Sal dominated forest**	12.75	13.63	15.72	0.88	6.9	2.09	15.33	2.97	23.29
**Mixed forest**	109.41	134.93	183.92	25.52	23.3	48.99	36.3	74.51	68.1
**Crop land**	139.15	117.21	66.89	-21.94	-15.76	-50.32	-42.93	-72.26	-51.92
**Developed area**	5.22	4.25	5.31	-0.97	-18.58	1.06	24.94	0.09	1.72
**A lower part of ACA**									
**Water bodies**	0.29	0.28	0.27	-0.01	-3.44	-0.01	-3.57	-0.02	-6.89
**Barren area**	5.23	4.39	3.97	-0.84	-16.06	-0.42	-9.56	-1.26	-24.09
**Grassland**	18.19	15.27	14.57	-2.92	-16.05	-0.7	-4.58	-3.62	-19.9
**Mixed forest**	86.96	95.88	99.95	8.92	10.25	4.07	4.24	12.99	14.93
**Crop land**	21.28	15.84	12.56	-5.44	-25.56	-3.28	-20.7	-8.72	-40.97
**Developed area**	0.54	0.83	1.17	0.29	53.7	0.34	40.96	0.63	116.66
