# Supplementary material for: Land use/land cover changes in the central part of the Chitwan Annapurna Landscape, Nepal
Source: PeerJ. 2022 May 20;10:e13435. doi: 10.7717/peerj.13435 (PMC9126145; doi:10.7717/peerj.13435)
Supplement: Supplemental Information 3 [file peerj-10-13435-s003.pdf]

Table S3 Error matrix resulting from classifying test pixels Accuracy assessment on the basis of ground truthing points (Land cover 2000).

Land cover	Water bodies	Barren land	Grass land	Riverine forest	Sal forest	Crop land	Developed area	Mixed forest	User's total	User's accuracy (%)
Water bodies	18	1	0	1	0	2	0	0	22	81.81
Barren area	0	11	0	0	0	0	0	4	15	73.33
Grass land	0	1	29	1	1	2	0	3	37	78.37
Riverine forest	0	0	0	27	0	1	0	3	31	87.09
Sal forest	0	0	1	0	64	5	0	6	76	84.21
Crop land	0	1	4	1	8	115	3	7	139	82.73
Developed area	0	0	1	0	0	4	21	1	27	77.77
Mixed forest	2	1	1	3	7	14	5	120	153	78.43
Producer total	20	15	36	33	80	143	29	144	500	
Producer's accuracy (%)	90	73.33	80.5	81.8	80	80.41	72.41	83.3		
