# Supplementary material for: Land use/land cover changes in the central part of the Chitwan Annapurna Landscape, Nepal
Source: PeerJ. 2022 May 20;10:e13435. doi: 10.7717/peerj.13435 (PMC9126145; doi:10.7717/peerj.13435)
Supplement: Supplemental Information 4 [file peerj-10-13435-s004.pdf]

Table S4 Error matrix resulting from classifying test pixels Accuracy assessment on the basis of ground truthing points (Land cover 2010).

Land cover	Water bodies	Barren land	Grass land	Riverine forest	Sal forest	Crop land	Developed area	Mixed forest	User's total	User's accuracy
Water bodies	10	1	0	1	0	1	0	0	13	76.92
Barren area	0	8	0	0	0	0	0	1	10	80
Grass land	0	0	12	1	1	1	0	1	16	75
Riverine forest	0	0	0	10	0	1	0	2	13	76.92
Sal forest	0	0	1	0	64	7	0	5	77	83.11
Crop land	1	1	1	1	8	135	3	12	162	83.33
Developed area	0	0	0	0	0	2	14	3	19	73.68
Mixed forest	2	1	1	1	7	15	4	160	191	83.77
Producer total	13	11	15	14	80	162	21	184	500	
Producer's accuracy	76.92	72.73	80	71.4	80	83.3	66.67	86.95		
