# Supplementary material for: Land use/land cover changes in the central part of the Chitwan Annapurna Landscape, Nepal
Source: PeerJ. 2022 May 20;10:e13435. doi: 10.7717/peerj.13435 (PMC9126145; doi:10.7717/peerj.13435)
Supplement: Supplemental Information 5 [file peerj-10-13435-s005.pdf]

Table S5 Error matrix resulting from classifying test pixels Accuracy assessment on the basis of ground truthing points (Land cover 2020).

**Class**	**Water**	**Barren area**	**Grass land**	**Riverine forest**	**Sal forest**	**Crop land**	**Developed area**	**Mixed forest**	**User total**	**User's accuracy (%)**
Water bodies	45	2	2	1	0	0	0	0	50	90
Barren area	5	41	2	0	0	0	2	0	50	82
Grassland	2	1	51	2	1	2	0	4	63	80.95
Riverine forest	1	2	3	55	1	1	0	4	65	84.61
Sal forest	0	2	2	2	117	2	1	3	129	90.69
Cropland	2	1	2	5	0	93	5	1	109	85.32
Developed area	0	2	1	0	0	5	55	2	65	84.62
Mixed forest	0	8	6	0	3	8	5	122	152	80.26
Producer total	55	59	69	65	122	111	68	136	683	
User accuracy (%)	81.18	69.49	76.11	84.61	95.9	83.78	80.88	89.7		
